# Trace Element Levels in Serum Are Potentially Valuable Diagnostic Markers in Dogs

**DOI:** 10.3390/ani10122316

**Published:** 2020-12-07

**Authors:** Yolanda Cedeño, Marta Miranda, Inmaculada Orjales, Carlos Herrero-Latorre, Maruska Suárez, Diego Luna, Marta López-Alonso

**Affiliations:** 1 Department of Animal Pathology, Faculty of Veterinary, Campus Terra, Universidade de Santiago de Compostela, 27002 Lugo, Spain; yoli19mp@hotmail.com (Y.C.); dluna@doctor.com (D.L.); marta.lopez.alonso@usc.es (M.L.-A.); 2 Faculty of Veterinary Medicine, Universidad Central del Ecuador, EC170521 Quito, Ecuador; 3 Department of Anatomy, Animal Production and Clinical Veterinary Sciences, Faculty of Veterinary, Campus Terra, Universidade de Santiago de Compostela, 27002 Lugo, Spain; inma.orjales@gmail.com (I.O.); maruska.suarez@usc.es (M.S.); 4 Rof-Codina Veterinary Teaching Hospital, Faculty of Veterinary, Campus Terra, Universidade de Santiago de Compostela, 27002 Lugo, Spain; 5 Research Institute on Chemical and Biological Analysis, Analytical Chemistry, Nutrition and Bromatology Department, Faculty of Sciences, Campus Terra, Universidade de Santiago de Compostela, 27002 Lugo, Spain; carlos.herrero@usc.es

**Keywords:** dog, trace elements, biochemical parameters, serum, inductively coupled plasma-mass spectrometry (ICP-MS)

## Abstract

**Simple Summary:**

Determining trace element concentrations in serum can provide useful information about some diseases. Furthermore, a single blood sample can nowadays be used to determine trace element profiles by multielement techniques such as inductively coupled plasma-mass spectrometry. Along with other routinely determined biochemical parameters, trace element profiles provide information about the pathogenesis, diagnosis, and prognosis of diseases, and enable evaluation of the suitability of trace element supplementation as an adjunct therapy. In this study, we evaluated the suitability of using trace element profiles, together with other routinely determined biochemical parameters, in dogs suffering different diseases, as diagnostic markers in routine clinical practice.

**Abstract:**

The objective of this study was to obtain information about the role of trace element imbalance in the pathogenesis of certain diseases in dogs and to evaluate the suitability of trace element profiling as an additional tool in the diagnosis. Serum trace element concentrations (copper, molybdenum, selenium and zinc) were measured in a cohort of healthy (control) dogs (n = 42) and dogs affected by hepatic (n = 25), gastrointestinal (n = 24), inflammatory/infection (n = 24), and renal (n = 22) diseases. These data were analyzed together with data on basic biochemical parameters (alanine aminotransferase, alkaline phosphatase, blood urea nitrogen, creatinine, albumin, globulin, and glucose) by using chemometric techniques. The chemometric analysis revealed distinctive association patterns between trace elements and biochemical parameters for each clinical disorders. The findings provide clear evidence for the important role of trace elements in disease, particularly in relation to acute phase reactions, with serum copper providing an indirect measurement of ceruloplasmin (positive acute-phase protein) and serum selenium and zinc acting as negative acute phase reactants. Molybdenum may also be a suitable marker of incipient renal disease. Thus, the analysis of trace element profiles, by multielement techniques, in a single serum sample would be a valuable additional tool for the diagnosis of certain diseases.

## 1. Introduction

Trace elements are essential micronutrients that play a vital role in many physiological and biochemical processes. As cofactors for multiple key enzymes, they are involved in antioxidant defense, immune response, redox signaling, wound healing, and regulation of gene expression, among other functions [1]. Trace element requirements are well defined both in humans [2] and domestic animals, including livestock and pets [3,4], and there is an almost century-long record of success and safety in food and feed fortification, with proven efficacy for preventing specifically related diseases [5]. Nonetheless, mineral deficiencies remain widespread worldwide.

Trace element deficiencies/disorders have been more widely studied in humans than in animals. The World Health Organization [6] considers that more than 2 billion people worldwide suffer from vitamin and mineral deficiencies, particularly in developing countries, but also in developed countries, affecting people of all ages, as well as certain risk groups. In most cases, trace element deficiencies and imbalances are clinically silent and do not cause specific diseases, but act as exacerbating factors in infectious and chronic diseases [5]. In recent years, a growing number of publications have reported trace element deficiencies and imbalances in various clinical disorders and particularly in critical illnesses, including cardiac, hepatic and renal failure, cancers, inflammatory diseases, and infection, among others [7,8,9,10,11,12,13,14]. In critically ill patients, oxidative stress can lead to the development of secondary tissue damage and organ failure, and serum levels of trace elements that contribute to antioxidant defense, particularly selenium (Se) and zinc (Zn), are typically low during critical illnesses [14]. Moreover, the magnitude of the inflammatory response following systemic inflammation is inversely correlated with plasma levels of most micronutrients [9], so that low micronutrient levels probably indicate an increased probability of organ damage and mortality. However, there are still many knowledge gaps and it is unclear whether the changes in plasma levels during critical illness simply reflect the acute-phase response (cellular shifts), a deficiency (alterations of absorption or intake), reduced availability (altered protein-binding), or enhanced metabolism [14].

Although information about the effects of trace element deficiencies is very scarce in veterinary medicine, there is evidence for an association between mineral imbalances and some pathological disorders. For example, Se and Zn deficiencies have been described in dogs suffering from diarrhea, irrespective of the etiology [15]; low levels of Se have been associated with a higher incidence of neoplasms and allergies [16]; increased serum concentrations of copper (Cu) and decreased serum concentrations of iron (Fe) and Zn have been found in dogs infected with *Hepatozoon canis* [17] and *Rangelia vitalii* (Apicomplexa: Piroplasmorida) [18]; and increased whole blood manganese (Mn) concentrations have been reported in dogs with primary hepatitis [19] and epilepsy [20].

The findings of a recent study by our research group in a cohort of dogs representative of a local population in NW Spain showed significant variations in the main trace elements (Cu, Se, Zn, and molybdenum [Mo]) in individuals suffering from different pathologies, relative to healthy control animals [21]. These preliminary findings suggested that trace element determination in serum could provide useful information about the pathogenesis of some diseases. Further investigation of serum concentrations of trace elements, together with those of other biochemical parameters, as routine markers of liver and renal function or inflammation, may be useful for routine clinic. In this sense, may provide valuable information (i) regarding the pathogenesis, as identification of symptoms or alterations related to trace element imbalance, due to e.g., oxidative damage; (ii) disease prognosis, as preventing complications and (iii) may also enable evaluation of the suitability of trace element supplementation as an adjunct therapy. In the present study, we used chemometric techniques to analyze the serum concentrations of trace elements and biochemical data from the same cohort of dogs used in the aforementioned study. The study objective was to obtain information about the role of trace element imbalances in the pathogenesis of the diseases considered and to evaluate the suitability of trace element profiling as an additional tool in disease diagnosis.

## 2. Material and Methods

### 2.1. Animals and Sample Collection

Chemical and biochemical data were obtained from 137 dogs attended between November 2015 and April 2017 in the Rof-Codina Veterinary Teaching Hospital, Faculty of Veterinary Medicine, University of Santiago de Compostela (north-west Spain). Data collection was carried out according to Directive 2010/63/EU on the protection of animals used for scientific purposes [22], and the trial complied with the Spanish legislation on animal care (RD 53/2013, 1 February 2013) [23]. The procedures used were supervised by the Bioethics Committee of the Rof-Codina Veterinary Teaching Hospital, University of Santiago de Compostela (Spain).

Detailed information on the dogs is described in a previously published paper [21]. In the present study, 137 dogs for which a clear and definitive diagnosis was possible, and basic biochemical analytical data were also available were selected from the initial sample of 187 dogs used in the previous research. The dogs were classified into five groups according to the pathologies suffered: healthy controls (n = 42; both males and females dogs, belonging mainly to the service-learning program of the animal shelter, attending the hospital for castration procedures), hepatic disease (n = 25; including chronic and vascular hepatic disorders), gastrointestinal disease (n = 24; acute and chronic disorders), inflammatory disease-infection (n = 24; including infectious and immune-mediated diseases), and renal disease (n = 22; acute and chronic renal disorders). All animals were adult (age range, 1.2 to 11.7 years) and no statistically significant differences were found between groups (*p* > 0.05). The male/female proportion was similar in all the groups. The remaining 50 dogs from the previous study (suffering cardiorespiratory, dermatological, neurological, and oncological pathologies) were not included in the present study due to the low number of samples in each group and/or the absence of complete biochemical data.

### 2.2. Sample Preparation and Biochemical and ICP-MS Analysis

Blood samples were obtained from the cephalic vein and were centrifuged at 3000 rpm for 5 min. Biochemical analysis was performed within 1–2 h of extraction, whereas for trace element determination by ICP-MS the samples were stored at −20 °C until analysis of all samples. 

Blood serum samples were analyzed to determine the following biochemical parameters: alanine aminotransferase (ALT), alkaline phosphatase (ALP), blood urea nitrogen (BUN), creatinine (CREA), albumin (ALB), globulin (GLOB), and glucose (GLU). For all samples, serum determinations were carried out using a Catalyst One analyzer (IDEXX Laboratories, Inc., Westbrook, ME, USA), which utilizes dry slide technology and is validated for this type of analysis [24]. The analysis was performed following the manufacturer’s instructions and included automatic software updates, cleaning, and quality control procedures (Quality Control Material, QCM; VetTrol control plus diluent, IDEXX Laboratories). The data obtained were also compared against the reference ranges for each variable (Table 1).

For ICP-MS analysis, serum samples were acid digested, and the concentrations of trace elements (cobalt [Co], chromium [Cr], Cu, Fe, Mn, Mo, nickel [Ni], Se, and Zn) and toxic elements (arsenic [As], cadmium [Cd], mercury [Hg] and lead [Pb]) were subsequently determined in the supernatant, by ICP-MS (Agilent 7700x ICP-MS system; Agilent Technologies, Tokyo, Japan). The sample collection procedure, serum preparation, and trace element determination by ICP-MS are described in detail in the aforementioned paper [21].

### 2.3. Data Analysis

All statistical analyses were carried out using the chemometric-statistical package Statgraphics Centurion XVIII, ver. 18.1.12 (Statistical Graphics, Rockville, MD, USA). The distribution of the biochemical data was checked using the Kolmogorov–Smirnov (K–S) test, and non-normally distributed data were log-transformed. The influence of the pathology on trace elements was evaluated by using a one-way ANOVA and post-hoc analysis. Data were also compared to the reference ranges. All differences were considered significant at *p* < 0.05.

For the chemometric procedures, the data used in this study were the concentrations of 12 metals (Co, Cr, Cu, Fe, Mn, Mo, Ni, Se, Zn, As, Hg, and Pb), determined by ICP-MS in the serum samples from the 137 selected dogs in the five groups described above, and the concentrations of 7 biochemical variables (ALT, ALP, BUN, CREA, ALB, GLOB, GLU), measured in the same samples. The results of preliminary assays were considered together with the results of a previous study in which Cu, Mo, Se, and Zn proved potentially useful markers for different diseases [21] to select the data set finally used in the study. The final data set comprised an *X*_137x11_ data matrix in which the files were the 137 selected dogs in the five groups described above, while the columns were the values of these 4 metals considered as valuable markers plus the 7 measured biochemical variables. 

Two multidimensional unsupervised display chemometric techniques were applied to study the latent relationships in the *X*_137x11_ data matrix. The first, Hierarchical Cluster Analysis (HCA), is a multidimensional display chemometric procedure that groups the samples or variables into clusters according to their similarity, evaluated in terms of the distance between each. In this case, the similarity between samples or variables was estimated using the squared Euclidean distance as a similarity measurement, and the Ward method (a hierarchical agglomerative procedure) was used to produce clusters [25]. The final clusters obtained with this technique can be visualized by graphical representation of the sample or variable clusters in the form of a dendrogram (a tree diagram used to display the arrangement of clusters produced by HCA). The second technique, Principal Component Analysis (PCA), is also a chemometric display technique, commonly used together with HCA, thus enabling examination of the data set in a reduced dimension while preserving as much information as possible [26]. PCA transforms the original data matrix *X*_137x11_ into a product of two matrices, one of which contains information related to the variables (score-matrix) and the other of which contains information related to the loading variables (loadings-matrix). When the number of principal components (PC) selected for visualization of the data set is lower than the number of original variables (n = 11), PCA provides an important simplification of the original data matrix X. 

The original data set was autoscaled prior to the application of both unsupervised chemometric HCA and PCA techniques, to prevent any influence due to the different magnitudes of the original variables. Autoscaling is a pretreatment procedure in which each value is subtracted from the mean value of the variable and divided by the corresponding standard deviation. The final result is a new set of variables, containing exactly the same information as the original variables, but of similar size, and all with zero mean and unit variance [27].

## 3. Results

### 3.1. Biochemical Parameters

**Figure 1:**
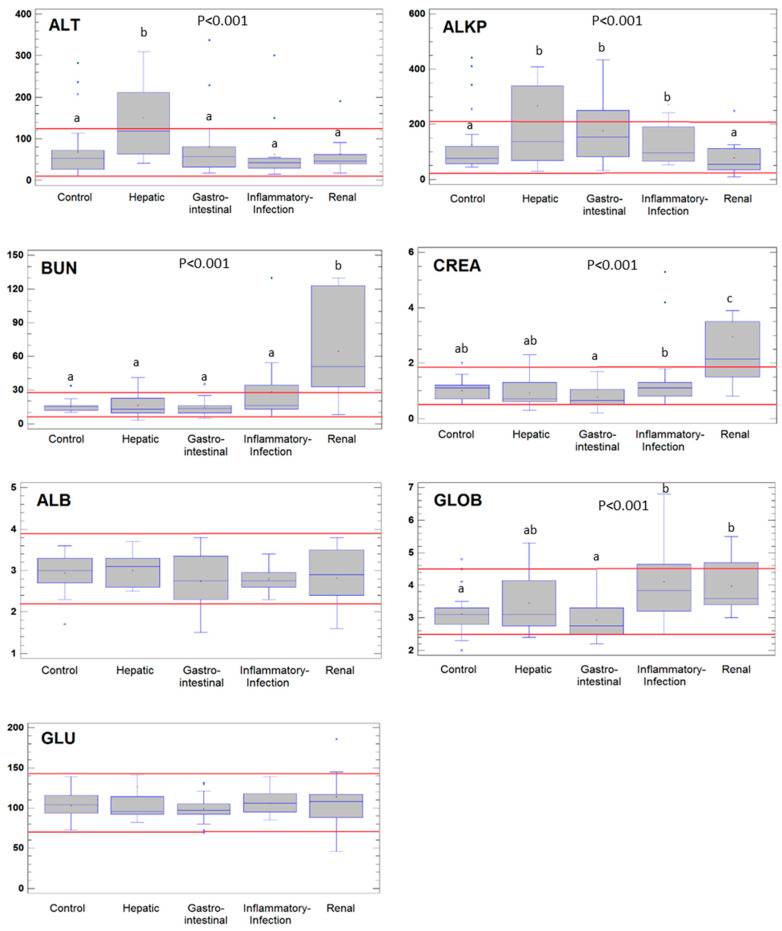
Box-and-whisker plot showing the concentrations of alanine aminotransferase (ALT) (U/L), alkaline phosphatase (ALP) (U/L), blood urea nitrogen (BUN) (mg/dL), creatinine (CREA) (mg/dL), albumin (ALB) (g/dL), and globulin (GLOB) (g/dL), and glucose (GLU) (mg/dL) in the serum samples from healthy control dogs and from dogs suffering from hepatic, gastrointestinal, inflammatory-infection and renal diseases. The red lines represent the upper and lower limits of the reference range for each variable. The horizontal blue line within the box denotes the median value of the variable; the red cross denotes the mean value; and the lower and upper boundaries of the box represent the first and third quartiles (thus, the box is the interval covering the middle 50% of the values); whiskers are drawn from the edges of the box to the highest and lowest values (except for values unusually far away from the box). In this case, the outliers, i.e., points more than 1.5 times the interquartile range (box width) above or below the box, are indicated by blue squares. Different letters indicate a statistically significant difference between groups (*p* < 0.05).

The basic biochemical parameters evaluated in the dogs in this study are presented in Figure 1 as box-whisker plots for each biochemical variable and group (control group and hepatic, gastrointestinal, inflammatory-infection, and renal disorders/diseases). 

Overall, the biochemical parameters are within the reference ranges (marked with red lines in each plot). As expected, statistically significant differences were observed in the dogs suffering from different disorders. ALT was significantly higher in dogs suffering from hepatic disorders and was above the upper normal range in more than 57% of the dogs. ALP was significantly higher in the groups affected by hepatic, gastrointestinal, and inflammatory-infection diseases than in the control group; 43 and 33% of dogs in the groups affected by hepatic and gastrointestinal diseases had ALP values above the normal range. BUN and CREA were significantly higher in the group affected by renal diseases, in which 86 and 54% of the dogs had values above the normal range for these parameters respectively. GLOB concentrations were significantly higher in the dogs suffering from hepatic, inflammatory-infection, and renal diseases; in the two last groups, 37 and 30% of individuals had GLOB concentrations above the physiologically normal range. Finally, no statistically significant differences were observed between the control and the other groups of dogs in relation to ALB or GLU.

### 3.2. Chemometric Analysis Showing the Relationship between Trace Element and Biochemical Parameters

**Figure 2:**
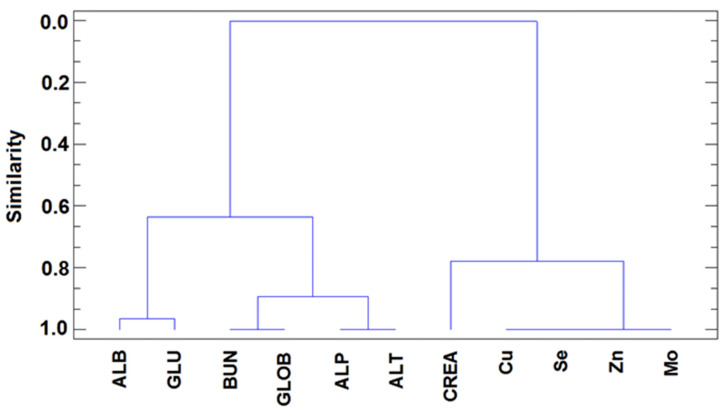
Dendrogram resulting from the hierarchical cluster analysis (HCA) (squared Euclidean distance and Ward agglomerative method) of data from the healthy control dogs.

To establish the relationships between the variables considered, HCA was applied to the *X*_137x11_ data matrix for the different groups. In all cases, the similarity between variables was calculated from the squared Euclidean distance between each, and the clusters were obtained by Ward agglomerative method. Various patterns were observed in different groups. The dendrogram obtained after application of HCA to the data from control dogs (see Figure 2) revealed two main, well-separated clusters. On the one hand, all of the parameters indicative of adequate liver function in healthy dogs are closely associated, and within them, three well-defined subclusters were identified: ALP and ALT (indicative of liver damage and cholestasis; in this case the absence of these), ALB and GLU (indicative of adequate metabolism when above the lower range) and BUN and GLOB (within the normal values indicative of the absence of disease). On the other hand, all trace elements (Cu, Mo, Se, and Zn) were closely associated and a relatively short distance from CREA (which in normal healthy individuals indicates muscle metabolism). 

**Figure 3:**
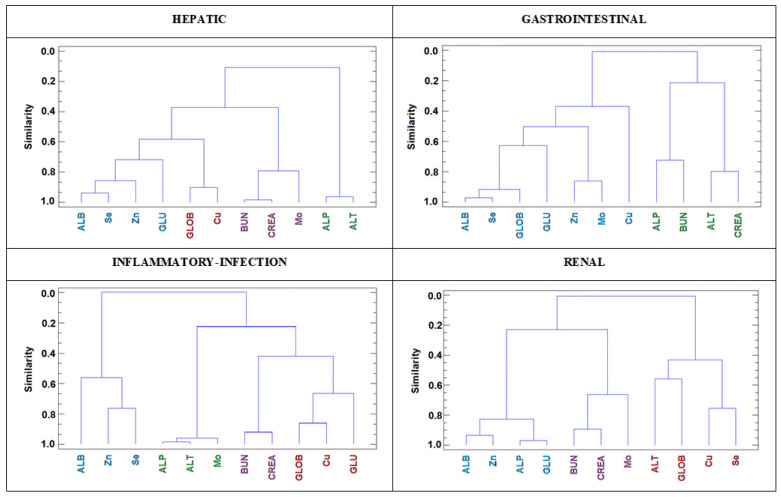
Dendrogram resulting from HCA (squared Euclidean distance and Ward agglomerative method) of the data from different groups of dogs affected by hepatic, gastrointestinal, inflammatory-infection, and renal diseases.

The results obtained by applying the same HCA procedure to the other four groups affected by different pathologies showed very different associations between trace elements and biochemical parameters for the dogs in these groups (Figure 3). The most outstanding relationships between trace elements and biochemical variables are summarized as follows: 

(i) Cu was closely associated with GLOB in the pathological disorders in which these proteins were elevated, namely hepatic, inflammatory-infection and renal diseases; in addition, Cu and GLOB were associated with GLU in the group affected by inflammatory disease-infection (reflecting a high level of GLU consumption in these individuals) and with Se in the group affected by renal disorders (Se is regulated by a renal homeostatic mechanism). 

(ii) Mo was closely associated with BUN and CREA in the groups suffering from renal and hepatic diseases; this association was expected as Mo is regulated by renal homeostatic mechanism, and in both groups, BUN and CREA clearance by the kidney could be affected. The close association of both ALP and ALT with Mo in the group affected by inflammatory disease-infection was also remarkable.

(iii) Both Se and Zn were closely associated with each other and with ALB (and to a lesser extent with GLU) in all groups, possibly indicating a role for these parameters in acute phase reactions. 

Finally, (iv) it is also worth noting that the dendrograms reveal close associations between biochemical parameters indicative of liver damage (ALP and ALT), renal function (BUN and CREA), liver function (ALB and GLU), and inflammatory challenge (GLOB; except here in the renal patients, in which the elevated GLOB possibly indicates a mechanism to compensate for the free ALB in urine).

**Figure 4:**
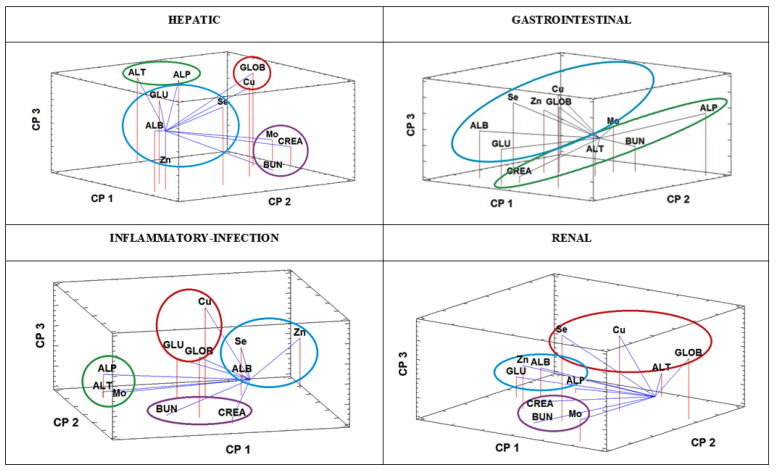
Loading plot of the variables in the space of the first three principal components obtained by PCA for the different groups of dogs affected by hepatic, gastrointestinal, inflammatory-infection, and renal diseases, representing 74.9, 65.0, 65.1, and 68.1% of the total variance, respectively.

Although most of the latent associations between variables revealed by HCA are logical and well defined, the relationships between variables were also studied by applying a second display chemometric technique as a verification procedure. Thus, PCA was also performed on the data on the four groups classified according to pathology. The results for the associations between trace elements and biochemical variables were evaluated in the loading plots of the variables in the space defined by the first three principal components. The loading plots for the four groups of diseases are presented in Figure 4 (accounting for 74.9%, 65.0%, 65.1%, and 68.1% of the total variance in data for hepatic, gastrointestinal, inflammatory-infection, and renal diseases, respectively). The PCA revealed the same associations between variables as those detected by HCA. For each of the diseases considered, the same variable association was demonstrated by both display chemometric techniques (see associations indicated by the same color codes in Figure 3 and Figure 4). The results were therefore verified by the consistent findings of two different chemometric techniques based on different mathematical premises.

## 4. Discussion

The results of the present study in a heterogeneous sample of dogs suffering from a wide variety of pathologies at different stages, in which a quite high level of experimental noise was expected, enabled us to confirm our previous findings [21] indicating that trace elements play a significant role in the pathogenesis of certain diseases and to confirm our hypothesis that trace elements together with other ordinary markers of disease could provide valuable information regarding the diagnosis and prognosis of some diseases in dogs. 

As previously stated, Cu serum concentrations were significantly higher in dogs affected by hepatic disorders and inflammatory disease-infection [21]. The study findings demonstrate that Cu is associated with GLOB in both of these groups, suggesting some connection with ceruloplasmin. Ceruloplasmin is an α2-glycoprotein that is considered one of the major positive acute-phase proteins in dogs [28]. It plays an important role in protecting host tissues from toxic oxygen metabolites released from phagocytic cells during inflammatory states [29]. It is also involved in Cu transport and antioxidant defense [30], the latter by inhibiting Cu ion-stimulated formation of reactive oxidants and the scavengers H_2_O_2_ and superoxide. Canine serum ceruloplasmin levels are increased during infection, inflammation, and trauma [17,18,31]. The increase is greater and becomes evident earlier than in humans, peaking at about two times normal values on the fourth day after surgery [31]. Measurement of this protein in dogs provides valuable information on the inflammatory status to veterinary clinicians [32] and is useful for monitoring the treatment of canine leishmaniasis [28]. If serum Cu is confirmed to be a reliable indicator of ceruloplasmin, it would be of diagnostic value in this type of inflammatory disease. Moreover, the close association between GLU and both Cu and GLOB also appears to support the role of Cu during inflammation. Glucose consumption is known to be enhanced during infection [33,34]. This so-called “stress hyperglycemia” results from the release of counter-regulatory hormones (glucagon, cortisol, and epinephrine), which oppose the action of insulin. They are released as part of the physiological “stress” response to infection [35]. 

In the present study, Cu (together with Se) was also related to GLOB in the group of dogs affected by renal disorders. In dogs, chronic kidney disease is associated with chronic inflammation (even in stable patients), which has been directly related to an increased risk of periodontal and cardiovascular disease [36]. As blood UREA and CREA only indicate a decrease of >75% in renal functional mass, there is a need for markers that enable early detection of renal damage and increased risk of complications [37]; in this respect, changes in serum Cu concentrations (as stated above) may provide valuable information. Interestingly, GLOB levels were also related to ALT in the group affected by renal disorders. In humans, serum ALT levels in patients with pre-dialysis chronic kidney disease tend to decrease in proportion to the progression of chronic kidney disease, and serum ALT levels are even lower in patients undergoing hemodialysis [38,39,40,41]. Factors that may be involved in reducing ALT serum levels in patients with chronic kidney disease and undergoing hemodialysis include lower pyridoxine serum levels, higher homocysteine levels, and hemodilution due to fluid retention [42]. 

In our previous study, serum Mo concentrations were found to be significantly enhanced in dogs suffering from renal disorders [21]. This was expected, as Mo is regulated by a renal homeostasis mechanism [43], and the finding is consistent with the close association between Mo and the kidney function markers BUN and CREA observed in the present study in the dogs affected by renal disorders. Information on Mo concentrations in serum of renal patients is very scarce. A recent study in dogs and cats with chronic interstitial nephritis demonstrated that less Mo was excreted in urine than in healthy animals [44]. In humans, it has been suggested that high serum Mo concentrations may contribute to dialysis-related bone disease in patients requiring long term hemodialysis, as massive Mo accumulation causes joint deformity and arthritis [45]. In the present study, Mo was also closely associated with BUN and CREA in hepatic patients. Although BUN and CREA are within the adequate range in the group of dogs affected by hepatic disorders, the association with Mo may indicate an early effect on kidney function. Chronic liver disease is responsible for a significant number of physiological changes that affect the circulation and kidney perfusion, which significantly affect serum concentration of CREA [46]. BUN may be decreased in animals suffering hepatic insufficiency [47], and CREA is not an early marker of kidney disease [48]. Biomarkers of kidney damage require further evaluation in the chronic liver disease population, as earlier diagnosis and implementation of currently established beneficial therapies seem may potentially reduce the severity of kidney damage and increase survival [46]. The present findings indicate that serum Mo together with BUN and CREA (even within the reference ranges) could provide useful information about incipient changes in the kidney, warranting further evaluation. Both chemometric techniques confirmed that ALP and ALT were closely associated with Mo in the dogs affected by inflammatory disease-infection. However, as far we are aware there is no information available in the scientific literature that would allow us to discuss the implication of this association. 

Finally, Se and Zn were closely associated with ALB. Albumin is a key somatic/plasmatic protein with a myriad of important physiological effects and is essential in any basic healthy biochemical profile. Quantitative changes in serum ALB concentrations represent an important indicator of the presence of disease or its progression or improvement. The significance of serum ALB levels is limited to varying degrees of hypoalbuminemia, as, except for cases of acute dehydration, hyperalbuminemia does not occur. In clinical medicine, hypoalbuminemia is generally not the result of a single mechanism. Causes of hypoalbuminemia include malnutrition, maldigestion and/or malabsorption syndrome, liver failure, protein-losing enteropathy, nephrotic syndrome, and also inflammation and infection, in which ALB is considered a negative acute-phase protein. Acute-phase proteins are a class of proteins whose plasma concentrations increase (positive acute-phase proteins) or decrease (negative acute-phase proteins) in response to inflammation and other acute physiological processes within the acute phase reactions [49]. The physiological role of decreased synthesis of ALB and other negative acute-phase proteins, such as selenoprotein P, is generally to save amino acids to produce “positive” acute-phase proteins more efficiently.

Together with negative acute-phase proteins, the levels of some circulating trace elements such as Zn and Se are known to decrease significantly during the acute phase reaction [49]. Hepatic Se metabolism has been shown to be increasingly disturbed during the acute phase reaction [50], thus negatively affecting serum Se status by insufficient biosynthesis of the central Se transport and the storage protein selenoprotein P; as a result, regular Se metabolism and Se transport is severely interrupted. Moreover, the hypoxia associated with severe disease has also been associated with a general decrease in selenoprotein P expression [51] and decreased Se export from hepatocytes. On the other hand, the decrease in Zn serum levels observed during acute phase reactions appears to be associated with the decreased hepatocytic secretion of ALB, as this is the main Zn transporter within the bloodstream; however, it is also possible that downregulation of the Zip-14 transporter [52] and synthesis by pro-inflammatory cytokines in the liver of metallothionein [53], a key molecule in the intracellular metal ion binding capacity, may also decrease serum Zn values [49]. Decreased concentrations of Se and Zn in these patients is very important as both elements are cofactors for essential enzymes: Se is essential for the catalytic activity of glutathione peroxidase, which protects against membrane lipid peroxidation, while Zn is a cofactor in zinc superoxide dismutase, which also combats oxidative stress [54]. In view of these roles, adequate trace element status is of particular importance in critically ill patients. Some authors propose that the reduction of these trace elements may deplete circulating antioxidants leading to elevation of reactive oxygen species and thereby exacerbating the severity of illness [51,55]. For this reason, monitoring the levels of Se and Zn in serum, together with ALB (negative acute phase protein) and other positive acute phase proteins, such as C reactive protein, may provide valuable information for evaluating critically ill patients. 

## 5. Conclusions

The study findings although preliminary, relating to a cohort of dogs suffering a wide variety of pathological disorders, clearly indicate those trace elements play an important role during disease, and trace element concentrations in serum could provide valuable information regarding the diagnosis and prognosis of some diseases in dogs. This is particularly true in relation to acute phase reactions where serum Cu proved to be an indirect measurement of ceruloplasmin (positive acute-phase protein), while serum Se and Zn were found to be negative acute phase reactants. Molybdenum may also be a suitable marker of incipient renal disease. Considering that trace element concentrations can nowadays be easily measured in many diagnostic laboratories (by multielement techniques such as ICP-MS), trace element profiling in serum could support the diagnosis made with other markers and also improve the prognosis of the associated risk (e.g., Se and Zn could be used as indirect measures of the antioxidative potential represented by glutathione peroxidase and superoxide dismutase). However, further research is still needed to precisely establish the sensibility, specificity, diagnostic accuracy of trace elements when used in the diagnosis of the different diseases. Finally, as trace element deficiencies are associated with an increased risk of morbidity and mortality, future research should be directed towards investigating the potential benefits of antioxidant and trace element supplementation in patients suffering from serious diseases.

## Figures and Tables

**Table 1:** Reference ranges for biochemical variables.

Variable	Reference Range	Dry Chemistry Method
ALT	10–125 U/L	LDH (without P5P)
ALP	23–212 U/L	pNNP + AMP + Mg
BUN	7–27 mg/dL	Urease
CREA	0.5–1.8 mg/dL	Enzymatic colorimetric
ALB	2.2–3.9 g/dL	Bromocresol green
GLOB	2.5–4.5 g/dL	calculation
GLU	70–143 mg/dL	Glucose oxidase, POD

LDH: lactate dehydrogenase; P5P: pyridoxine-5-phosphate; pNNP: *p*-nitro-phenylphosphate; AMP: 2-amino-2-myethyl-1-propanol; POD: peroxidase.
